# Neonatal Exposure to Bisphenol A and Reproductive and Endocrine Alterations Resembling the Polycystic Ovarian Syndrome in Adult Rats

**DOI:** 10.1289/ehp.0901257

**Published:** 2010-04-22

**Authors:** Marina Fernández, Nadia Bourguignon, Victoria Lux-Lantos, Carlos Libertun

**Affiliations:** 1 Instituto de Biología y Medicina Experimental, Buenos Aires, Argentina; 2 Facultad de Medicina, Universidad de Buenos Aires, Buenos Aires, Argentina

**Keywords:** bisphenol A, GnRH pulsatility, ovary, PCOS, sex hormones

## Abstract

**Background:**

Bisphenol A (BPA), an endocrine disruptor, is a component of polycarbonate plastics, epoxy resins, and polystyrene. Several studies have reported potent *in vivo* effects, because BPA behaves as an estrogen agonist and/or antagonist and as an androgen and thyroid hormone antagonist.

**Objectives:**

We investigated the effects of neonatal exposure to BPA on the reproductive axis in adult female Sprague-Dawley rats.

**Methods:**

Female rats were injected subcutaneously, daily from postnatal day 1 (PND1) to PND10 with BPA in castor oil at 500 μg/50 μL [BPA500; ~ 10^−4^ M, a dose higher than the lowest observed adverse effect level (LOAEL) of 50 mg/kg], 50 μg/50 μL (BPA50), or 5 μg/50 μL (both BPA50 and BPA5 are doses lower than the LOAEL), or castor oil vehicle alone. In adults we studied *a*) the release of gonadotropin-releasing hormone (GnRH) from hypothalamic explants, *b*) serum sex hormone levels, and *c*) ovarian morphology, ovulation, and fertility.

**Results:**

Neonatal exposure to BPA was associated with increased serum testosterone and estradiol levels, reduced progesterone in adulthood, and altered *in vitro* GnRH secretion. Animals exposed to BPA500 had altered ovarian morphology, showing a large number of cysts. Animals exposed to BPA50 had reduced fertility without changes in the number of oocytes on the morning of estrus, whereas animals exposed to BPA500 showed infertility.

**Conclusions:**

Exposure to high doses of BPA during the period of brain sexual differentiation altered the hypothalamic–pituitary–gonadal axis in female Sprague-Dawley rats. These results have the potential to link neonatal exposure to high doses of BPA in rats with the development of polycystic ovarian syndrome. Studies of doses and routes of administration more consistent with human exposures are needed to determine the relevance of these findings to human health.

According to the U.S. Environmental Protection Agency (EPA), an endocrine disruptor is “an exogenous agent that interferes with the synthesis, secretion, transport, binding, action, or elimination of natural hormones in the body that are responsible for the maintenance of homeostasis, reproduction, development and/or behavior” (Kavlock et al. 1996). Bisphenol A (BPA), one of these compounds, is one of the highest volume chemicals produced worldwide, with > 6 billion pounds produced each year. BPA is a constituent of polycarbonate plastics and epoxy resins used in the food industry and in dentistry. The polymer bonds hydrolyze at high temperatures and release BPA. BPA can be ingested by humans; detectable amounts of BPA have been found in food cans, microwave containers, human saliva after treatment with dental sealants, and polycarbonate bottles (Vandenberg et al. 2007). More elevated levels of BPA have been found in neonates and children. Infants can be exposed to BPA through different sources; as noted by Vandenberg et al. (2007), detectable levels of BPA in breast milk (0.28–0.97 ng/mL and 1.1 ng/mL) have been reported in two different studies, as well as in human colostrum (1–7 ng/mL) and in polycarbonate baby bottles. BPA has also been found in medical devices (Calafat et al. 2009). Edginton and Ritter (2009) estimated the daily exposure for newborns and children at 3 and 6 months of age, under different feeding scenarios, finding exposures that ranged from 0.25 μg/kg/day in breast-fed newborns to 8.3–13 μg/kg/day in 6-month-old infants fed commercial formula or other beverages using polycarbonate bottles.

Although *in vitro* assays have suggested that BPA is a weak environmental estrogen, it has also been shown to antagonize the effects of estrogens, androgens, and thyroid hormones; act through nongenomic pathways; and influence enzyme activity or receptor expression (Wetherill et al. 2007). *In vivo* effects also have been reported based on studies using a wide range of doses, animal models, and end points. The current U.S. EPA reference dose for BPA (50 μg/kg/day) was calculated by dividing the lowest observed adverse effect level (LOAEL; 50 mg/kg/day) by 1,000 (Diamanti-Kandarakis et al. 2009). In some studies, low doses are considered those less than the LOAEL. Effects of BPA can be influenced by species, strain, dose, and time of exposure, and adult animals exposed to BPA show effects that are reversible when the exposure ceases (Richter et al. 2007). In contrast, perinatal/neonatal exposures produce “organizational” effects [effects resulting from exposure during organ development and continuing through puberty that may result in persistent alterations of the affected systems (Richter et al. 2007)] in different strains of rats (Adewale et al. 2009; Durando et al. 2007; Fernández et al. 2009; Kato et al. 2003; Khurana et al. 2000; Moral et al. 2008; Patisaul et al. 2006; Ramos et al. 2003; Rubin et al. 2001).

Gonadotropin-releasing hormone (GnRH) is a hypothalamic decapeptide critical for normal mammalian reproduction. It acts on gonadotropes to stimulate synthesis and release of luteinizing hormone (LH) and follicle- stimulating hormone (FSH) (Pawson and McNeilly 2005). GnRH secretion is pulsatile, and its frequency varies over the female menstrual cycle and during development. Increases in plasma gonadotropins follow the GnRH peaks, and disruption in the normal pulsatile secretion causes reproductive disorders in humans, such as polycystic ovarian syndrome (PCOS) (Marshall et al. 2001). PCOS is considered the most common endocrine abnormality in reproductive-age females—occurring in 4–8% of women—and is characterized by elevated serum LH, testosterone, and insulin; low or normal levels of FSH; abnormalities of estrogen secretion; and premature pubic hair growth (pubarche). There is heterogeneity in these alterations among women with polycystic ovaries, and in some individuals these characteristics may change over time. Women with PCOS are at increased risk of developing insulin resistance, diabetes, endometrial cancer, and anovulatory infertility. Thus, this syndrome is characterized by metabolic and reproductive disorders (Marshall et al. 2001).

Previously, we have shown that neonatal BPA exposure alters pituitary function in postnatal day 13 (PND13) and adult female rats, both *in vitro* and *in vivo* (Fernández et al. 2009). In the present study we continued to explore the effects of neonatal exposure to BPA on other reproductive parameters in female Sprague-Dawley rats. We analyzed GnRH release from hypothalamic explants, sex hormone levels in serum, ovarian morphology, ovulation, and fertility.

## Materials and Methods

### Animals

Studies were performed according to protocols for animal use, approved by the Institutional Animal Care and Use Committee [IByME-CONICET (Instituto de Biología y Medicina Experimental, Consejo Nacional de Investigaciones Científicas y Técnicas]. Animals were treated humanely and with regard for alleviation of suffering.

Sprague-Dawley rats (200–250 g body weight) from the IByME colony, established from Charles River stock in 1985, were maintained under a controlled 12-hr light/dark cycle and temperature conditions. They were housed in steel cages with wood shavings as bedding material and given free access to commercial laboratory chow (Gepsa Feeds, Grupo Pilar S.A, Córdoba, Argentina) and tap water in glass bottles. Phytoestrogen concentration in the food was not tested, but we assumed that all animals were exposed to the same levels, because food intake was equivalent among groups (Fernandez MO, Bourguignon NS, Lux-Lantos VAR, Libertun C, unpublished data). In our experiments, phytoestrogens were constant in the diet and BPA was tested at different doses, so we cannot exclude the possibility of some interaction between BPA and phytoestrogens (Charles et al. 2007).

Pregnant females were housed singly, and on the day of birth (PND1), pups in each litter were reduced to eight. From PND1 to PND10, female pups received daily subcutaneous (SC) injections of BPA (Aldrich, Milwaukee, WI, USA) in castor oil, as previously described (Fernández et al. 2009): 50 μg/50 μL (BPA50; dose range, 6.2–2.5 mg/kg), 500 μg/50 μL (BPA500; dose range, 62.5–25.0 mg/kg), 5 μg/50 μL (BPA5; dose range, 0.62–0.25 mg/kg), or castor oil vehicle alone (control). Each litter included one female pup in each exposure group, so that a given litter included one control, one BPA5, one BPA50, and one BPA500; this allowed us to control for possible effects due to dam variability. The four remaining pups in each litter were untreated males; they were kept until weaning but were not used for this study.

The highest BPA dose used in this study (500 μg/μL) has been used by others in this and other strains of rats (Khurana et al. 2000; Patisaul et al. 2006, 2007); because it is above the LOAEL, we consider it “high dose.” The 50 μg/50 μL and 5 μg/50 μL doses were below the LOAEL and are therefore referred to as “low doses.” Although the SC route is not a typical route of exposure in humans (Richter et al. 2007), we chose it based on a report from Taylor et al. (2008) that showed that during the neonatal period, oral and nonoral administration of BPA give the same internal active dose in rodents because of the low liver enzyme activity in neonatal rodents.

We previously determined that adult BPA500 animals had irregular estrous cycles, with high prevalence of estrus (Fernández et al. 2009); therefore, studies were performed with all animals in estrus, determined by vaginal smears. For all these studies, animals were used at 4–5 months of age.

### Serum sex hormone levels

Serum estradiol, testosterone, and progesterone were determined in trunk blood by radioimmunoassay (RIA) as previously described (Bianchi et al. 2004; Silveyra et al. 2007), using specific antisera kindly provided by G.D. Niswender (Colorado State University, Fort Collins, CO, USA) after ethyl ether extraction. Labeled estradiol and progesterone were obtained from Perkin-Elmer (Wellesley, MA, USA), and testosterone was from New England Nuclear (Boston, MA, USA). Assay sensitivities were 11.3 pg (estradiol), 12.5 pg (testosterone), and 500 pg (progesterone). Intra-and interassay coefficients of variation were 6.8% and 11.7% for estradiol, 7.1% and 12.15% for progesterone, and 7.8% and 12.3% for testosterone, respectively.

### GnRH release from hypothalamic explants

Studies were performed *in vitro* as described previously (Fernández et al. 2009). One hypothalamic explant from each animal group (control, BPA50, BPA500) was used for each experiment. After 30 min of preincubation, the medium from each flask was renewed at 9-min intervals for 6 hr and stored at −20°C for GnRH measurement (RIA).

GnRH pulses were identified and interpulse intervals (IPIs) determined by Cluster8 analysis (Veldhuis and Johnson 1986) using Pulse_XP software (http://mljohnson.pharm.virginia.edu/home.html). We used a 2 × 2 cluster configuration and a *t*-statistic of 2 for the upstroke and downstroke to maintain false-positive and false-negative error rates < 10% (Martinez de la Escalera et al. 1992). The experiment was repeated six times, with animals from BPA500, BPA50, and control groups in each experiment (total of 6 hypothalami/group).

### Ovarian morphology

To evaluate changes in the ovarian structure, we collected ovaries from rats in each group (control, BPA50, and BPA500). Some of the ovaries were randomly selected for weighing after laparotomy, and others were immediately fixed in 5% neutral buffered formaldehyde for histological examination. Sections cut from paraffin-embedded ovaries were stained with hematoxylin and eosin to count preovulatory follicles, corpora lutea, antral follicles, and atretic follicles using a light microscope as described by Parborell et al. (2005).

### Ovulation and fertility

On the morning of estrus, ovaries were removed from seven animals in each treatment group, and ovulation was evaluated by microscopic examination of the oviducts (Silveyra et al. 2007). To determine fertility, we placed female rats from each experimental group with a male of known fertility (one female from each treatment: control, BPA5, BPA50, and BPA500, four in total, with one male). When females were determined to be pregnant, they were separated and housed individually. We counted the number of pups on the day of birth (PND1).

### Statistical analysis

Results are expressed as mean ± SE, and values are considered significant at *p* < 0.05. Data were analyzed by one-way analysis of variance (ANOVA) (Statistica, version 5; Statsoft Inc., Tulsa, OK, USA) with a Student-Newman-Keuls posttest and transformed when the test for homogeneity of variances so required.

## Results

### Sex hormone levels and GnRH IPIs in adult female rats

**Figure 1:**
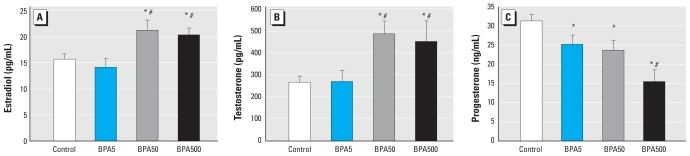
Neonatal exposure to BPA alters sex hormone levels in serum of adult female rats. (*A*) Serum estradiol (*n* = 7). (*B*) Serum testosterone (*n* = 9). (*C*) Serum progesterone (*n* = 9). *p < 0.05 compared with control, and ^#^*p* < 0.05 compared with BPA5, as determined by ANOVA.

We first determined sex hormone serum levels in BPA-treated animals. Adult BPA500 and BPA50 animals had higher levels of testosterone and estradiol, and all BPA groups showed lower levels of progesterone than controls, although BPA500 was the most affected group (Figure 1).

**Figure 2:**
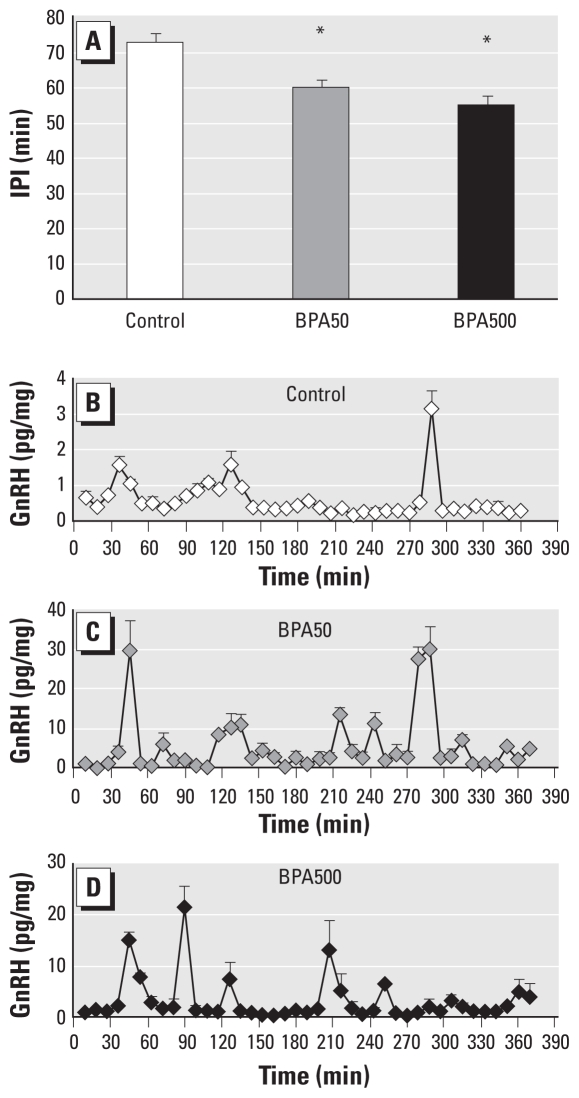
Neonatal exposure to BPA diminishes GnRH IPI in adult female rats, as determined by GnRH release from hypothalamic explants *in vitro* (*n* = 6 per treatment). (*A*) GnRH IPI. Representative pulsatility patterns of control (*B*), BPA50 (*C*), and BPA500 (*D*) treatment. *p < 0.05 compared with control, as determined by ANOVA.

Researchers have shown that alterations in sex hormone levels could modify GnRH/LH pulsatility (Marshall et al. 2001). We therefore measured this parameter *in vitro* in BPA50 and BPA500 hypothalami, because these were the treatment groups in which more alterations had been observed. Adult female rats neonatally exposed to BPA presented alterations in GnRH secretion, as demonstrated by a decrease in the IPI (Figure 2A), in agreement with results from our previous study (Fernández et al. 2009). Representative patterns of GnRH secretion are shown in Figure 2B–D.

### Morphological alterations in the ovary

**Figure 3:**
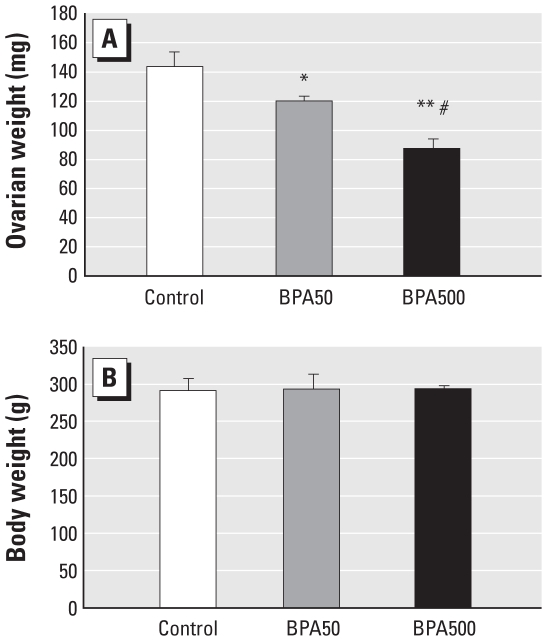
Neonatal exposure to BPA alters ovarian weight without changing body weight in adult rats. (*A*) Ovarian weight. (*B*) Body weight of the same animals. *p < 0.05 compared with control, **p < 0.005 compared with control, and ^#^*p* < 0.05 compared with BPA50, as determined by ANOVA (*n* = 6 per treatment).

Alterations in sex hormone levels and GnRH pulsatility could be consistent with alterations at the ovarian level, so we studied some ovarian parameters in control, BPA50, and BPA500 animals, because these two BPA-exposed groups revealed alterations in GnRH IPI. Macroscopic observation showed that ovaries from the BPA-exposed animals were smaller than those from controls, as confirmed by ovarian weight (Figure 3); there were no differences in body weight.

**Figure 4:**
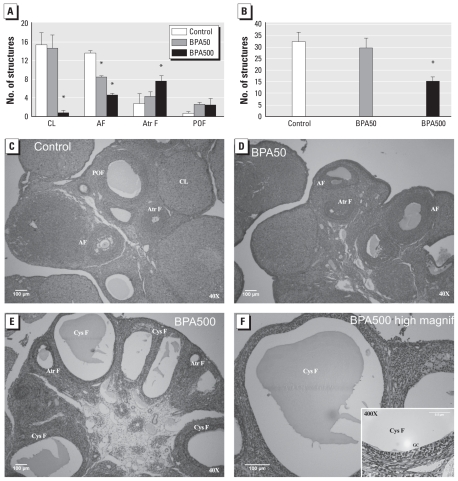
Neonatal exposure to BPA alters ovarian histology in adult female rats. Ovaries from control and BPA-treated animals were stained with hematoxylin and eosin and viewed under a light microscope. Abbreviations: AF, antral follicles; Atr F, atretic follicles; CL, corpus luteum; GC, granulosa cell; POF, preovulatory follicles. (*A*) Number of ovarian structures by type of structure. (*B*) Total number of structures by treatment. (*C–F*) Representative slides from each treatment. (*C*) Control. (*D*) BPA50. (*E*) BPA500. (*F*) A higher magnification of (*E*), showing a cystic follicle in detail; the inset shows the thin granulosa layer and nondetectable theca in the follicle. *p < 0.05 compared with control, by structure type in (*A*) and by treatment in (*B*), as determined by ANOVA (*n* = 5).

Histological analysis of the ovaries demonstrated a large number of cysts in BPA500 animals, which were comparable with high-dose animals reported in other animal models of PCOS (Sotomayor-Zárate et al. 2008). We also determined the number of corpora lutea, antral follicles, atretic follicles, and preovulatory follicles in each group. As shown in Figure 4A, adult animals neonatally exposed to BPA500 showed a lower number of corpora lutea and a higher number of atretic follicles, many of which were cystic. We define “cystic follicles” as anovulatory follicles with a thin granulosa layer (two to three layers) and nondetectable theca (Figure 4E,F). Both BPA-treated groups showed lower numbers of antral follicles. As shown in Figure 4B, BPA500 animals had a lower total number of structures, in agreement with the decrease in ovarian weight. Figure 4C–F shows representative sections from each group.

### Alterations in fertility

**Figure 5:**
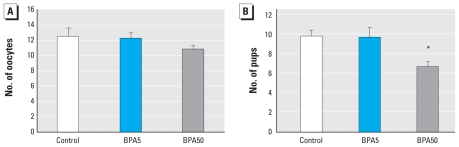
Neonatal exposure to BPA reduces fertility in adult females. (*A*) Number of oocytes in the ampulla of the oviduct. (*B*) Number of pups delivered. *n* = 7 per group. BPA500 females did not present any oocytes or deliver any pups. *p < 0.05 compared with control, as determined by ANOVA.

We also investigated whether neonatal exposure to BPA impaired ovulation and/or fertility. In controls and all BPA-exposed groups, we counted the number of oocytes in the ampulla of the oviduct on the morning of estrus, and the number of pups delivered after being mated with a male of known fertility. BPA500 animals did not ovulate or deliver any pups. BPA50 and BPA5 animals presented the same number of oocytes as controls (Figure 5A). In fertility studies, BPA5 animals delivered the same number of pups as controls, whereas BPA50 animals delivered significantly fewer pups, indicating subfertility (Figure 5B).

## Discussion

We previously showed that neonatal exposure to BPA had profound effects on the organization of the hypothalamic–pituitary unit in female rats (Fernández et al. 2009). In the present study, we were interested in determining whether exposure to BPA during the neonatal period (which is important for brain sexual differentiation) had other effects on the reproductive axis.

We found that exposure to 500 and 50 μg/day BPA accelerated GnRH pulse frequency in hypothalamic explants from adult rats. We previously reported that this exposure increased the number of GnRH peaks per hour (Fernández et al. 2009), and here we quantify this according to the length of the IPI, which is reduced. Many endocrine disorders are associated with alterations in GnRH pulsatility, and a very common disorder found in adult women, PCOS, is characterized by an increase in GnRH/LH pulse frequency and elevated LH/FSH index. An increase in GnRH/LH pulsatility could be a cause and/or consequence of alterations in sex hormone levels. In our model, we found elevated levels of serum estradiol and testosterone, and a decrease in progesterone. We hypothesize that the elevation in testosterone is driven by the increase in GnRH/LH pulse frequency, as has been reported in PCOS (Marshall et al. 2001). However, the reduction in progesterone could be causing the altered GnRH pulsatility, because progesterone is a well-known regulator of GnRH pulse frequency (Marshall et al. 2001). A decrease in serum levels of progesterone is another feature present in PCOS (Marshall et al. 2001). Interestingly, Takeuchi et al. (2004) found that serum BPA concentrations were higher in a small sample of women with PCOS. However, some researchers have speculated that elevated BPA is a consequence, and not a cause, of PCOS (Crain et al. 2008), because women with PCOS have higher circulating testosterone levels than do healthy women, and elevated androgen concentrations decrease BPA clearance (Diamanti-Kandarakis et al. 2009).

Because animals in the BPA500 and BPA50 groups showed extensive hormonal alteration in the gonadotropic axis, we next focused our attention on the ovary. Ovaries from animals neonatally exposed to BPA (50 or 500 μg/day) were smaller than those from controls, and ovaries from BPA500-treated animals were smaller than those from animals treated with BPA50. In addition, ovaries from BPA500 animals had fewer corpora lutea, fewer antral follicles, and more atretic follicles than did those from controls. In fact, many of the atretic follicles were cystic, and these cysts were very similar to those found in animal models of PCOS (Sotomayor-Zárate et al. 2008). BPA50 animals also had fewer antral follicles, but they did not show the profound alterations observed in high-dose animals. The alterations might be due to direct actions of BPA in the ovary or to indirect effects resulting from alterations in the hypothalamic–pituitary unit, or a combination of both.

Sotomayor-Zárate et al. (2008) described a decrease in the number of antral follicles and corpora lutea in animals neonatally exposed to estradiol valerate (EV; 0.1 mg on PND1). These authors demonstrated that the EV exposure provoked alterations in the normal development of the ovary, leading to the formation of multifollicular cysts, and they also described alterations to estrus cycles. In an earlier study, Kato et al. (2003) described cystic follicles in animals exposed daily to BPA (1 or 4 mg) from PND1 to PND10; they observed an absence of corpora lutea in animals exposed to 4 mg but not in animals exposed to 1 mg. In the present study, we found ovaries with cysts and absence of corpora lutea in animals exposed to 500 μg/day, whereas animals exposed to 50 μg/day presented some less severe alterations. Adewale et al. (2009) found that animals exposed to BPA from PND0 to PND3 at a dose of 50 mg/kg body weight per day developed ovarian alterations similar to those we observed here. However, they did not find altered activation of GnRH neurons, measured using Fos immunostaining after ovariectomy and hormone replacement. One difference between the studies is the technique used to measure activity in GnRH neurons: We measured the frequency of GnRH released by the hypothalamus in intact females and found alterations in all BPA groups tested. Another difference is the duration of the exposure; Adewale et al. (2009) treated animals for 4 days total, whereas we treated animals for 10 days (PND1–PND10).

So far, the results from the present study are the first to link neonatal exposure to high doses of BPA with development of PCOS-like abnormalities in adulthood. Further studies are needed to determine if rats neonatally exposed to BPA show other features associated with PCOS, such as insulin resistance, obesity, and neuronal and immune system alterations (Chapman et al. 2009; Sotomayor-Zárate et al. 2008). Rubin et al. (2001) found that perinatal exposure to BPA affected body weight in adult rats. Other authors have reported that long-term treatment of male mice with BPA altered the pancreatic content and secretion of insulin and resulted in postprandial insulinemia and insulin resistance (Alonso-Magdalena et al. 2006). In another study, Hugo et al. (2008) found that, *in vitro*, BPA inhibits the release of adiponectin (a key adipokine that protects humans from metabolic syndrome) from human adipose tissue. Other groups have reported that *in vitro* BPA affects adipocyte differentiation (Masuno et al. 2005) and increases expression of differentiation genes (Phrakonkham et al. 2008). These results support the hypothesis that perinatal exposure to BPA could affect metabolic parameters (Rubin and Soto 2009).

Regarding the mechanism by which BPA acts to produce the effects seen here, many hypotheses can be made. BPA has been shown to act through estrogen receptors (ERs) and through other mechanisms (Wetherill et al. 2007). Ovarian alterations observed in the present study mimic the results obtained with EV by Sotomayor-Zárate et al. (2008). In our study, the molar concentrations of BPA administered ranged between 10^−4^ M (BPA500) and 10^−5^ M (BPA5); the EV concentration used by Sotomayor-Zárate et al. (2008) was similar to the molar concentration of our medium dose (BPA50). According to Heisterkamp et al. (2004), who compared the potency of BPA and EV with the ERα, the median effective concentration (EC_50_) of BPA is higher than the EC_50_ of EV; therefore, the results of the present study and those of Sotomayor-Zárate et al. (2008) could suggest that both compounds are acting by the same mechanisms. However, the specific receptors through which BPA acts in the entire animal have been reported to be different depending on the tissue in question, because it has been shown that for a given cell type, the activity of BPA depends on the levels of different ER variants expressed (Wetherill et al. 2007). We also have to keep in mind that the outcomes we are describing are evident in adulthood and may be the result of an accumulation of alterations, rather than being outcomes triggered by a unique event.

Children can be exposed to BPA doses ranging from 0.2 μg/kg/day to 8.3–13 μg/kg/day (Edginton and Ritter 2009), which would correspond to a single dose of 10^−9^ M to 10^−8^ M. To calculate children’s exposure levels, we assumed exposure to a single dose of BPA to an infant whose body weight was 3.5 kg (PND5) or 7 kg (6 months old). Because the doses we used in the present study are much higher than the doses infants may be exposed to (~ 10^−5^ M vs. ~ 10^−9^ M), further research should be performed using doses that are more relevant to human exposure. We chose the SC route of exposure based on a report by Taylor et al. (2008) that shows this route is valid in neonates; however, we admit that oral administration would be more relevant to humans. In addition, some researchers do not support the conclusion that injections and oral administration produce similar internal exposures (Richter et al. 2007). Also, the experimental protocol we used—treating littermates with the different doses—could result in cross-contamination and is thus a potential limitation of the present study.

Finally, we studied ovulation and fertility in adult animals neonatally exposed to BPA. We found that animals exposed to BPA50 and BPA5 were comparable with controls regarding the number of oocytes in the oviduct on the first day of estrus, whereas BPA500 animals did not ovulate, in accordance with our previous observations (Fernández et al. 2009). In fertility studies we found that BPA50 females delivered a significantly smaller number of pups than did controls and that BPA500 animals were infertile. Varayoud et al. (2008) reported that neonatal exposure to BPA was associated with altered gene expression and hormone responsiveness in uterine stroma in adulthood, which could contribute to impaired fertility. However, other effects, such as effects on sex hormone levels during pregnancy or oocyte quality, could also contribute to reduced fertility. Indeed, oocytes are one of the longest lived, nonregenerating cells in the body and are subject to a lifetime of environmental exposures that are difficult to quantify (Crain et al. 2008). Further studies are needed to analyze effects of exposure to BPA on these parameters. For example, it would be interesting to treat the affected animals with pregnant mare’s serum gonadotropin and human chorionic gonadotropin to determine if the fertility of these animals can be rescued, or if there is also a uterine component underlying the infertility.

The results presented here support the hypothesis that neonatal exposure to high doses of BPA affects the reproductive axis in adulthood. We observed these changes in animals > 4 months of age, long after the exposure to the endocrine disruptor had ceased. We suggest that neonatal exposure to 500 μg BPA (a dose higher than the LOAEL) provoked irreversible alterations in the hypothalamic–pituitary–gonadal axis that led to anovulation and infertility, and that 50 μg BPA (a dose lower than the LOAEL) provoked more subtle alterations at the hypothalamic level that could lead to subfertility. The effects of lower BPA doses (more relevant to human exposure) should be studied further. The results presented here reinforce the notion that many diseases that appear in adulthood have their origin during development and that alterations in the normal ontogeny may result in irreversible “organizational” effects [effects that begin during prenatal development and continue through puberty, resulting in persistent alterations of the affected systems (Richter et al. 2007)] that may become evident only in adults.
